# *Pasteurellaceae* ComE1 Proteins Combine the Properties of Fibronectin Adhesins and DNA Binding Competence Proteins

**DOI:** 10.1371/journal.pone.0003991

**Published:** 2008-12-22

**Authors:** Lisa M. Mullen, Janine T. Bossé, Sean P. Nair, John M. Ward, Andrew N. Rycroft, Giles Robertson, Paul R. Langford, Brian Henderson

**Affiliations:** 1 Division of Microbial Diseases, UCL Eastman Dental Institute, University College London, London, United Kingdom; 2 Department of Biochemistry and Molecular Biology, University College London, London, United Kingdom; 3 Department of Pathology & Infectious Diseases, Royal Veterinary College, North Mimms, Hertfordshire, United Kingdom; 4 School of Crystallography, Birkbeck College, University of London, London, United Kingdom; 5 Molecular Infectious Disease Group, Department of Paediatrics, Imperial College London, St. Mary's Campus, Norfolk Place, London, United Kingdom; Charité-Universitätsmedizin Berlin, Germany

## Abstract

A novel fibronectin-binding protein from *Pasteurella multocida* (PM1665) that binds to the fibronectin type III_9-10_ modules via two helix-hairpin-helix motifs has recently been described [1]. This protein shares homology with competence-related DNA-binding and uptake proteins (ComEA and ComE) from Gram-positive and Gram-negative bacteria. Here, we show that recombinant PM1665 (now designated ComE1) also binds to DNA through the same helix-hairpin-helix motifs required for fibronectin-binding. This binding to DNA is non sequence-specific and is confined to double-stranded DNA. We have cloned and expressed ComE1 proteins from five members of the *Pasteurellaceae* in order to further investigate the function(s) of these proteins. When expressed as recombinant GST-fusion proteins, all of the homologues bound both to fibronectin and to double-stranded DNA. Inactivation of the gene encoding the ComE1 homologue in *Actinobacillus pleuropneumoniae* indicates major roles for these proteins in at least two processes: natural transformation, and binding of bacteria to fibronectin.

## Introduction

The *Pasteurellaceae* are a family of bacteria within the phylum proteobacteria that are predominantly mucosal colonists of man and animals. The family contains important human (*Haemophilus influenzae*, *Aggregatibacter (Actinobacillus) actinomycetemcomitans*, *Haemophilus ducreyi*) and animal (*Pasteurella multocida*, *Actinobacillus pleuropneumoniae*, *Mannheimia haemolytica*, *etc.*) pathogens as well as a range of commensal organisms [2]. As with all bacteria, colonisation of specific niches in host species is dependent on the selective binding of the microorganism to some host component(s). Bacterial molecules which allow such high affinity binding are termed adhesins and one of the most common host molecules for which adhesins have evolved is the essential, multifunctional and ubiquitous glycoprotein, fibronectin (Fn) [3], [4].

We know surprisingly little about the adhesins used by the *Pasteurellaceae* to colonise their human or animal hosts. In an attempt to identify genes coding for novel *Pasteurellaceae* adhesins we employed a functional genomic screening methodology, phage display. This identified a gene, *pm1665*, encoding a small Fn-binding protein from *P. multocida* that is 115 amino acids in length, with a predicted signal sequence and two predicted helix-hairpin-helix domains. Analysis of recombinant PM1665 revealed that it is a unique Fn-binding protein in that it binds to the cell binding domain of this glycoprotein, and specifically to the so-called type III (FnIII) domains FnIII_9-10_ [1]. Binding is of reasonably high affinity (approximately 100 nM). All other known bacterial Fn-binding proteins bind to the Fn type I N-terminal (heparin-, gelatin-binding) domain or to the C-terminal heparin binding domain of Fn. In addition to being a Fn-binding protein, we produced evidence (cell surface location and blocking of bacterial binding to Fn by an antiserum to PM1665) that PM1665 is likely to function as a bacterial adhesin. We were unable to generate *P. multocida* mutants with an inactivated gene encoding PM1665, so were not able to fully test this hypothesis.

Sequence analysis reveals that PM1665 has homology to the C-terminal region of the *Bacillus subtilis* DNA-uptake protein ComEA [5], as well as to the ComE proteins of *Neisseria gonorrhoeae* [6] Homologues are also identifiable in all of the whole genome sequences available for other members of the *Pasteurellaceae* [7]. The PM1665 homologue in *Haemophilus influenzae* (HI1008) has been designated ComE1 by Redfield et al. [8] on the basis of experimental evidence demonstrating that this gene is up-regulated almost 300-fold in cells that have been starved to induce competence. Hence, in this manuscript, PM1665 and homologous *Pasteurellaceae* proteins will be referred to as ComE1. As of yet, there is no evidence, based on mutation of the *comE1* gene, for the role of ComE1 in DNA binding or uptake in *H. influenzae* or other members of the *Pasteurellaceae*.

The sequence homology between the ComE1 proteins in members of the *Pasteurellaceae* and the well-characterised ComEA proteins in Gram-positive bacteria is confined to the two C-terminal helix-hairpin-helix (HHH) motifs and a 6-amino acid sequence (VNINTA) upstream of the first HHH domain. We have shown that these two HHH motifs plus the conserved 6-mer sequence are essential for binding of ComE1 from *P. multocida* to Fn [1].

Given that the HHH motif is indicative of DNA-binding proteins [9], [10] and the fact that both ComEA and ComE are DNA-binding proteins, an obvious question was whether ComE1 could also bind to DNA, in addition to the fibronectin binding activity already established [1]. We have now examined the ComE1 proteins from five members of the *Pasteurellaceae* and have demonstrated that they can all bind both Fn, via a unique mechanism, and double stranded DNA. Additionally, we have shown that ComE1 plays a major role in natural transformation in *A. pleuropneumoniae*– an unexpected concatenation of evolved functions.

## Materials and Methods

### Bacterial strains and plasmids

*H. influenzae* NCTC 8470/ATCC 9332 Pittman type D and *P. multocida* NCTC 10322/ATCC 43137 (pig isolate) were purchased from the National Collection of Type Cultures (London, UK) and cultured on chocolate agar or grown in Brain Heart Infusion (BHI) broth (Oxoid Ltd., Basingstoke, United Kingdom) aerobically at 37°C. BHI broth was supplemented with 10 µg/ml haemin and 2 µg/ml β-NAD (Sigma-Aldrich Co. Ltd. Poole, United Kingdom) in the case of *H. influenzae*. *A. pleuropneumoniae* serovar 15, strain HS143 was routinely cultured on either chocolate agar or BHI agar supplemented with 2 µg/ml NAD (BHI-NAD), or grown in either Columbia (Difco) or BHI-NAD broth, aerobically at 37°C. *A. actinomycetemcomitans* strain HK1651 (JP2 clone) was maintained on blood agar or grown in BHI broth at 37°C in a 5% CO_2_ atmosphere. *M. haemolytica* was maintained on blood agar or grown in BHI broth at 37°C. All strains used were clinical isolates. For expression of recombinant proteins, the GST-fusion expression vector pGEX6-P-1 (GE Healthcare) was used with either *Escherichia coli* Rosetta-gami™ DE3 (Novagen) or *E. coli* BL21 (DE3) as host strains.

### Cloning of the genes for homologues of ComE1

Oligonucleotides containing recognition sequences for the restriction enzymes, *Xho*I and *Eco*RI were designed to amplify the genes coding for ComE1 homologues from *H. influenzae*, *A. pleuropneumoniae*, *A. actinomycetemcomitans*, *M. haemolytica* and *M. succiniproducens* without their predicted signal sequences. The primer pairs used to amplify these genes from genomic DNA from each of the aforementioned bacteria are detailed in Table 1. The gene coding for Ms0826 from *M. succiniproducens* [11] was generously provided by Professor Sang Yup Lee (Korea Advanced Institute of Science and Technology). The PCR products obtained were then cloned into *Xho*I/*Eco*RI digested pGEX6-P-1 (GE Healthcare) and introduced into *E. coli* Rosetta gami (DE3) cells by chemical transformation.

**Table 1:** Oligonucleotides used to amplify genes encoding homologues of ComE1. Restriction sites are underlined.

*comE1* Homologue	Primer pairs
Hi1008	5′ CGGAATTCGAGGAAAAAGCGACAGA 3′
	5′ CCGCTCGAGTTAAAAGATTATACG 3′
APL_1406	5′ GGAATTCAAGCCTAATAATCCGCCC 3′
	5′ CCGCTCGAGTTATTCTAACGTGATG 3′
Aa1426	5′ CGGAATTCGCGGAAAAAGCG 3′
	5′ CCGCTCGAGTTATAAGGCGATACG 3′
MhORF35	5′ CGGAATTCCAAACCACTCAACCTACT 3′
	5′ CCGCTCGAGGAAAGTTAAATATGGAGC 3′
MS0826	5′CGGAATTCTTGACCACACTTTTTTTAAT 3′
	5′ CCGCTCGAGTTATAATTTTATCCGATCTTT 3′

### Expression and purification of recombinant proteins

For gene expression, positive clones were grown to log phase in Nutrient broth-2 containing 200 µg/ml of ampicillin at 30°C. Gene expression was induced with 1 mM isopropyl-ß-D-thiogalactopyranoside (IPTG) for 2 hours at 30°C. Cells were harvested and then resuspended and lysed for 30 minutes in 4 ml of B-PER protein extraction reagent (Pierce) containing 750 mM ammonium chloride and 50 µl of protease inhibitor cocktail (Sigma; Product number: P8465). The lysates were clarified by centrifugation at 15,000×*g* for 10 minutes, diluted 1∶1 in PBS and purified on a GSTrap column (GE Healthcare).

### Direct binding ELISA for measuring binding of recombinant proteins to Fn or DNA

Nunc maxisorb microtiter plates were coated overnight at 4°C with 100 µl aliquots of 100 µg/ml solutions of Fn (Sigma) in coating buffer (0.05 M carbonate–bicarbonate buffer, pH 9.6) or 10 µg/ml of chromosomal DNA from *P. multocida* or 10 µg/ml pUC19 DNA in Reacti-bind DNA coating solution (Pierce). Assays were performed as described previously [1] Briefly, recombinant GST-fusion proteins were added to wells coated with Fn or DNA, and the amount of GST-fusion proteins bound to wells determined by detection with an anti-GST antibody (GE Healthcare).

### Surface Plasmon Resonance (SPR)

The dissociation equilibrium constant (K_D_) for the interaction of recombinant ComE1 proteins (after removal of the GST-tag) with immobilized pUC19 DNA was determined by SPR using a BIAcore 3000 system (BIAcore AB International). DNA was biotinylated using the Biotin DecaLabel DNA Labeling kit (Fermentas) according to the manufacturer's instructions and immobilized on a SA sensor chip (BIAcore) via the interaction between the biotin on the DNA and the streptavidin on the sensor chip. Increasing concentrations of the analyte (ComE1) were flowed across both reference and DNA-coated flow cells in running buffer (PBS containing 0.05% Tween 20), at 25°C using a flow rate of 10 µl/min. Binding was determined by measuring the increase in resonance units after subtraction of the background response obtained from the reference flow cell.

### Competition ELISA

For inhibition ELISAs, recombinant GST-fusion proteins were pre-incubated for 1 h at 37°C with soluble Fn (Sigma), bacterial chromosomal DNA or pUC19 DNA. Reactions were then added to Fn-coated wells and bound rGST-ComE1 was detected with an anti-GST antibody as described above.

### Bacterial binding assays

The capacity of bacteria to bind to immobilised DNA or Fn was determined as described previously [1]. Briefly, triplicate wells of a Nunc Maxisorb microtitre plate were coated with 10 µg/ml of pUC19 DNA in Reacti-bind DNA coating solution (Pierce) overnight at room temperature or 10 µg/100 µl of Fn in coating buffer overnight at 4°C. Bacteria grown to log-phase or stationary phase were added to wells and allowed to bind for 1 hour at 37 C. Bound bacteria were removed from the wells by the addition of 100 µl of 0.25% trypsin and plated in triplicate. For inhibition assays, 100 µl of recombinant GST-ComE1 at a concentration of 25 µM were added to wells coated with DNA or Fn. The number of bacteria that bound to each well was then determined as described previously [1].

### Construction of the A. pleuropneumoniae mutant *ApΔcomE1*

To generate the deletion mutant of ComE1 in *A. pleuropneumoniae*, the *comE1* gene was replaced with the *Kan^R^* gene from plasmid pJMK30 [12]. Primers 5′-ACAAGCGGTTTCACCCATTCGGGTTTCTACG-3′ and 5′-CCTCCTCATCCTCTTCATCCTAAAAAAATCCGCTGAGCC-3′ were used to amplify a 1 kb flanking region upstream of *comE1*, primers 5′-GGCTCAGCGGATTTTTTTAGGATGAAGAGGATGAGGAGG-3′ and 5′-CAAGACGGTTCTCGCCTGTCATCTAAATCTAGGTACTAAAAC-3′ were used to amplify the *Kan^R^* gene, and 5′-GTTTTAGTACCTAGATTTAGATGACAGGCGAGAACCGTCTTG-3′ and 5′-ACAAGCGGTGTAGTTTCAGTCGTAGGCGCTG-3′ were used to amplify a 1 kb flanking region downstream of *comE1*. The forward primer for the 1 kb upstream flanking fragment and the reverse primer for the downstream 1 kb flanking fragment also included the uptake signal sequences (underlined) for *A. pleuropneumoniae*. These PCR products were joined together by subsequent PCR reactions. The resulting recombinant PCR product was used to transform *A. pleuropneumoniae* HS143 using natural transformation via the uptake signal sequences, as previously described [13]. Resulting *Kan* ^R^ colonies were confirmed to contain the *Kan* ^R^ gene in place of *comE1* by PCR and sequencing. To determine clearly that the mutation event only affected the desired gene and not other genes, *comE1* was cloned into the plasmid pMIDG311, a chloramphenicol resistant derivative of pJSK411 [14] containing the *A. pleuropneumoniae sodC* promoter upstream of a multiple cloning site, and introduced by conjugation into the *ApΔcomE1* mutant from *E. coli* S17 λ *pir*. PCR analysis of complemented mutants clearly indicated the presence of the *comE1* gene.

### Plate transformation assay

*A. pleuropneumoniae* HS143 and its isogenic mutant strain, *ApΔcomE1*, were tested for the ability to undergo natural transformation by plate assay [13]. We previously used genomic DNA from *A. pleuropneumoniae* serotype 1 carrying a *Kan* ^R^ insertion in the *sodC* gene (Ap1*sodC*::*Kan*) as donor DNA for transformation experiments [13]. However, the *ApΔcomE1* strain contains a *Kan* ^R^ insertion replacing the *comE1* gene. Therefore, it was necessary to generate donor DNA carrying a different selectable marker. To achieve this, we replaced the *Kan* ^R^ insertion in *sodC* in the plasmid pJSK333 [15] with a chloramphenicol resistance (*Chl* ^R^) cassette, and introduced this by natural transformation into the chromosome of *A. pleuropneumoniae* HS143 (Ap15*sodC*::*Chl*). The resulting *Chl* ^R^ colonies were screened by PCR to confirm the presence of the *Chl* ^R^ cassette within the *sodC* gene following homologous recombination. Subsequently, genomic DNA was prepared using the Qiagen mini-DNA kit and used as donor DNA for transformation.

### MIV transformation in the presence/absence of Fn

In order to determine if binding of soluble Fn to the surface of the bacteria would competitively inhibit natural transformation of *A. pleuropneumoniae* HS143, we used a modification of the MIV broth transformation assay previously described [13]. Briefly, bacteria were inoculated into BHI-NAD broth and incubated at 37°C with shaking until the OD_600_ reached 0.1, at which point 1.5 ml aliquots were centrifuged at 10,000 g for 5 min. The pellets were washed twice with MIV and finally resuspended in 1.5 ml MIV containing various concentrations of soluble Fn (from 0 to 300 µg/ml). Cultures were incubated at 37°C for 100 min to induce competence prior to addition of 1 µg DNA to appropriate samples. Cultures were then incubated for 25 min at 37°C, agitated gently on a roller, after which 10 U DNAse I were added. Two volumes of BHI-NAD were added, and the cultures were incubated for an additional 100 min prior to plating out dilutions on selective and non-selective agar.

## Results

### Binding of recombinant GST-ComE1 to DNA

**Figure 1:**
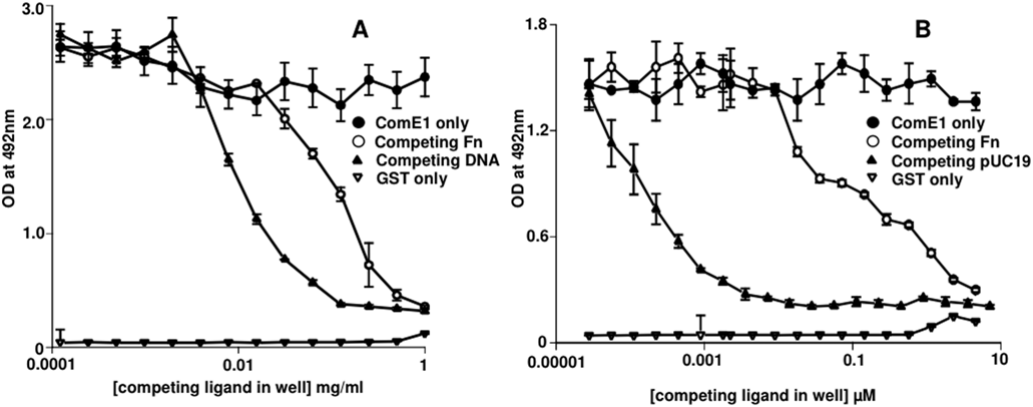
Binding of DNA to ComE1. (A) Inhibition ELISA to determine the capacity of Fn or genomic DNA isolated from *P. multocida* to block the binding of rGST-ComE1 from *P. multocida* to immobilised human serum Fn. The competing ligands were added at concentrations ranging from 1 mg/ml to 0.1 µg/ml. (B) Inhibition ELISA to determine the capacity of Fn or pUC19 DNA to block the binding of rGST-ComE1 from *P. multocida* to immobilised human serum Fn. Equimolar concentrations of Fn of pUC19 DNA were used to compete with immobilised Fn for binding to rGST-ComE1. Results are presented as the mean±SEM of quadruplicate wells and are representative of at least three experiments. The values for rGST-ComE1 refer to the binding of this protein to Fn-coated wells with no competing ligands.

**Figure 2:**
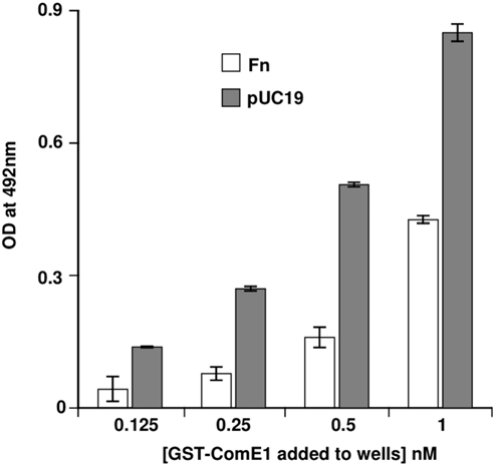
Binding of pUC19 to ComE1. Binding of 1 nM of recombinant GST-ComE1 from *P. multocida* to immobilised Fn or pUC19 DNA measured by direct binding ELISA. The data are presented as the mean±SEM of triplicate wells. The data shown are representative of three separate experiments.

**Figure 3:**
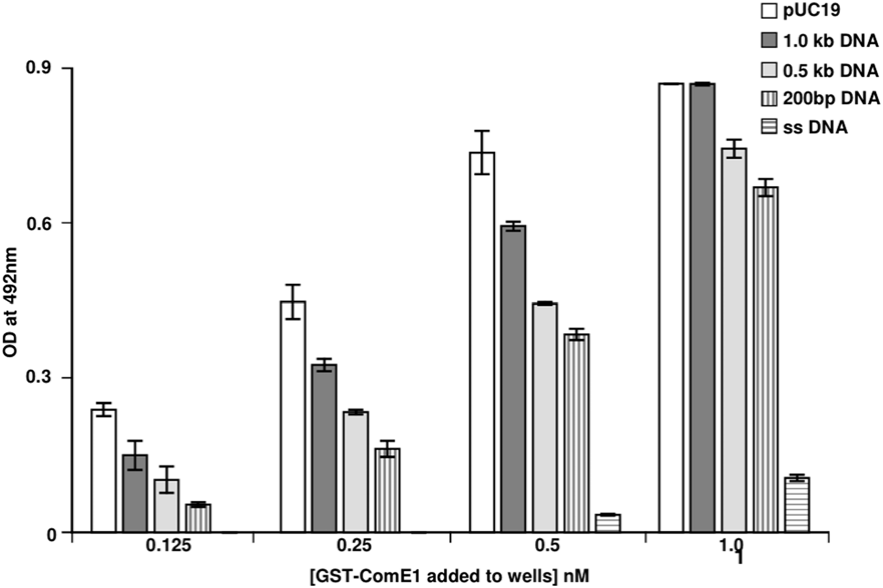
Binding of ComE1 to single-stranded DNA. Binding of 1 nM of recombinant GST-ComE1 from *P. multocida* to immobilised DNA fragments or single-stranded DNA (ssDNA) measured by direct binding ELISA. The data are presented as the mean±SEM of triplicate wells. The data shown are representative of three separate experiments.

**Figure 4:**
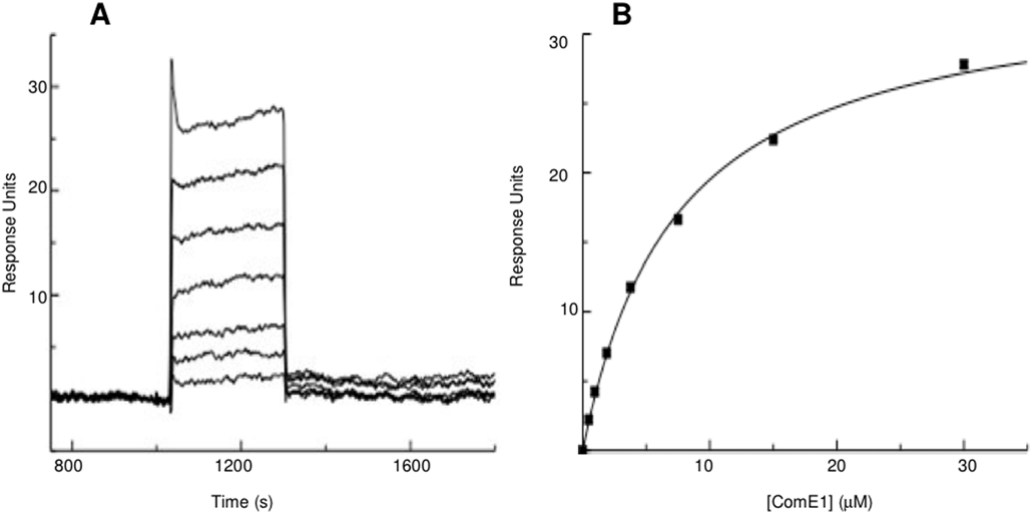
SPR analysis of binding of ComE1 from *P. multocida* to pUC19 DNA. [A] – pUC19 was immobilised on a SA sensor chip and increasing concentrations of ComE1 (1.1 µM, 2.2 µM, 4.4 µM, 8.5 µM, 17 µM, and 34 µM) were injected at a flow rate of 10 µl min^−1^ and the binding response recorded as response units (RU). Data are representative of three separate experiments. [B] – The equilibrium response for binding to Fn at each concentration of ComE1. Data are representative of three separate experiments.

The fact that ComE1 from *P. multocida* has sequence homology to ComEA DNA-binding proteins led to the hypothesis that this protein could have a role in DNA binding in addition to Fn binding. To test this hypothesis, the ability of recombinant GST-ComE1 to bind to immobilised DNA was tested by both competition and direct binding ELISA. In initial experiments, bacterial chromosomal DNA (isolated from *P. multocida*) was incubated with the GST-ComE1 prior to adding the fusion protein to Fn-coated wells. Soluble Fn was also used as a competitor as a positive control. The bacterial DNA inhibited the binding of GST-ComE1 to Fn indicating that not only does GST-ComE1 bind to DNA, but DNA appears to be a better inhibitor of the binding of the fusion protein to immobilised Fn than soluble Fn (Fig. 1a). However, this effect could be due to the fact that unequal molar concentrations of Fn and DNA were used as competitors. To circumvent the difficulties in accurately determining the molecular mass of the genomic DNA, subsequent experiments were performed using purified pUC19 as a source of DNA. The advantage of this approach was that this allowed the addition of known molar amounts of DNA to each well. When the competition ELISA was repeated using equimolar concentrations of Fn and DNA to compete with the GST-ComE1, DNA was again a much better competitor for binding to GST-ComE1 (Fig. 1b). These results were confirmed by a direct binding ELISA using wells coated with equimolar concentrations of either Fn or pUC19 DNA and measuring the relative amounts of GST-ComE1 bound (Fig. 2). However, these differences in observed binding could be due to multiple binding sites on the DNA molecules, as opposed to the two known FnIII_9-10_ binding sites on the Fn dimer. To investigate whether GST-ComE1 might be able to bind at multiple locations along the length of the DNA molecules, direct-binding ELISAs were used to test the binding of GST-ComE1 to equimolar concentrations of DNA fragments of varying length. Binding of GST-ComE1 to single-stranded (ss) DNA was also tested. Decreasing the length of DNA fragments used to coat microtitre plate wells resulted in a decrease in the amount of GST-ComE1 bound (Fig. 3) and there was no significant binding to ss DNA (Fig. 3). We have previously demonstrated that the interaction between GST-ComE1 and Fn has a K_D_ value of about 100 nM. Therefore, a quantitative measurement of the binding affinity of GST-ComE1 for pUC19 DNA was determined using surface plasmon resonance to allow direct comparison of the K_D_ values for the two interactions. Increasing concentrations of ComE1 were passed across a flow cell coated with pUC19 and the binding response recorded as changes in response units (RU) after subtraction of the binding response for the reference flow cell. A representative sensorgram is shown in Fig. 4A. The K_D_ of the interaction was calculated to be 7.3 µM±1.5 µM by analysis of the equilibrium binding data from two separate experiments (shown in Fig. 4B) using the Langmuir binding model.

### Identification of the DNA-binding site in P. multocida ComE1

**Figure 5:**
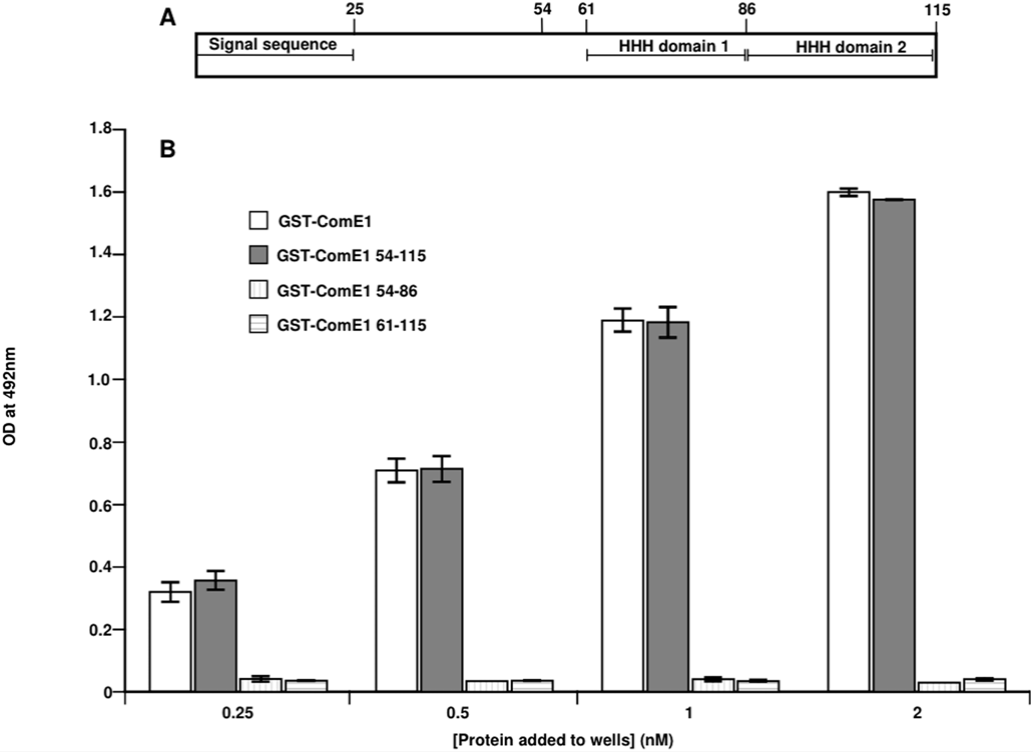
Schematic representation of the structural features of ComE1 (A). Binding of fragments of ComE1 from *P. multocida* (expressed as GST fusion proteins) to pUC19 DNA measured by direct binding ELISA (B). Increasing concentrations of rGST-ComE1 (open circles), the C-terminal 64 residues of ComE1 (closed circles), the two HHH domains of ComE1 (closed triangles) or a combination of the conserved VNINTA motif plus the first HHH domain (open triangles), were added to wells coated with Fn. Optical density values at 492 nm were converted to estimates of the concentration of bound protein by reference to a standard curve for each protein.

ComE1 from *P. multocida* binds to Fn via two C-terminal helix-hairpin-helix (HHH) domains, together with a highly conserved 6-mer sequence (VNINTA) located just before the first HHH domain [1]. In order to investigate which, if any, of these motifs are required for binding of ComE1 to DNA, three fragments of ComE1 were expressed as GST fusion proteins. The first fragment contained both of the HHH motifs (residues 61–115), the second fragment consisted of the conserved VNINTA(S) motif and the first HHH motif (residues 54–86), while the third fragment consisted of the entire C-terminal half of the protein i.e. the conserved VNINTA(S) motif plus both HHH motifs (residues (54–115). A direct binding ELISA was used to test the binding capacity of synthetic peptides consisting of the sequence of each of the HHH motifs. Neither of the individual HHH motifs bound to DNA (data not shown), nor did a combination of these two motifs, expressed as a GST-fusion protein (see Fig. 5). However, the GST-fusion protein consisting of the conserved VNINTA motif plus both helix-hairpin-helix motifs bound to DNA to a similar extent as mature rGST-ComE1 (Fig. 5). The VNINTA motif plus the first HHH motif did not bind to DNA (Fig. 5), indicating that, as for binding to Fn, all three regions (the VNINTA motif and both HHH motifs) are necessary for ComE1 binding to DNA.

### Effect of recombinant GST-ComE1 on binding of P. multocida to DNA

**Figure 6:**
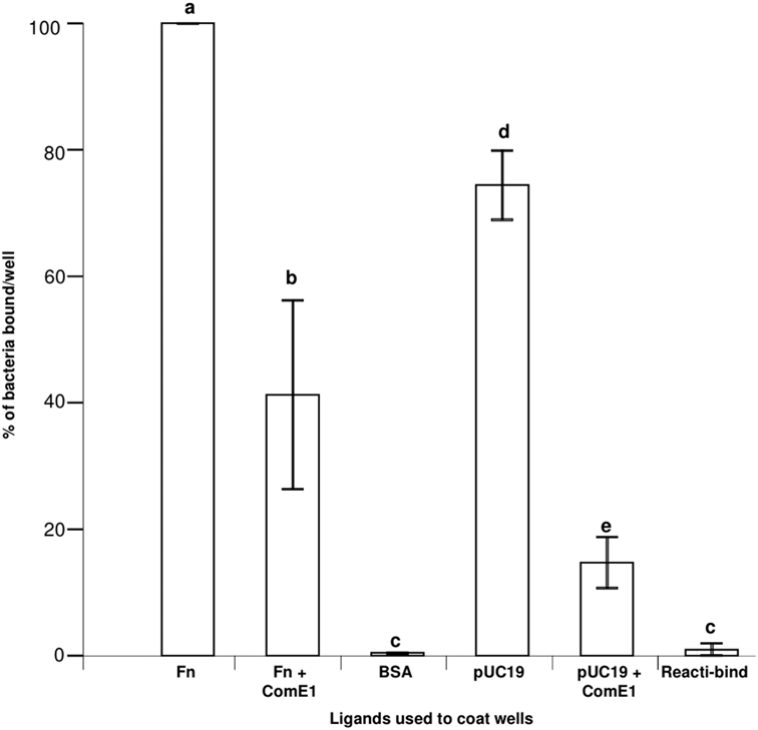
Binding of *P. multocida* to Fn or pUC19 pre-incubated with rGST-ComE1 from *P. multocida*. Values are expressed as percentage values of the binding to Fn. Bars with different letters are significantly different from each other (one-way ANOVA, P<0.001). Data are the mean±SEM of triplicate wells. Results are representative of three separate experiments.

It has recently been shown that recombinant *P. multocida* GST-ComE1 can block binding of *P. multocida* to Fn. To determine whether GST-ComE1 could have a similar effect on the binding of *P. multocida* to DNA, pUC19-coated wells were incubated with 25 µM of recombinant GST-ComE1 prior to the addition of *P. multocida* cells. Binding of *P. multocida* to Fn was also tested as a positive control. The number of *P. multocida* cells bound to immobilised pUC19 DNA was 80% of those bound to Fn and GST-ComE1 inhibited the binding of *P. multocida* to DNA by about 80% (Fig. 6).

### Presence of ComE1 homologues in other members of the Pasteurellaceae

**Figure 7:**
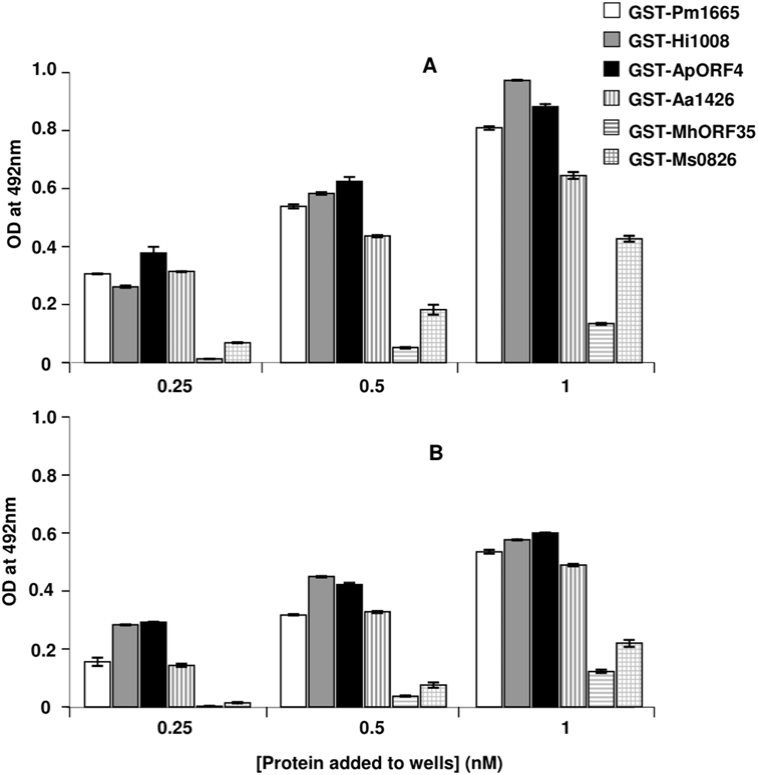
Binding of homologs of ComE1 from *P. multocida* (PM1665), *H. influenzae* (Hi1008), *A. actinomycetemcomitans* (Aa1426), *A. pleuropneumoniae* (APL_1406), *M. haemolytica* (MhORF35) or *M. succiniproducens* (Ms0826) expressed as GST-fusions to pUC19 DNA (A) or Fn (B) measured by direct binding ELISA. Optical density values at 492 nm were converted to estimates of the concentration of bound protein by reference to a standard curve for each protein.

Bioinformatic analysis had previously identified the presence of homologous proteins in members of the *Pasteurellaceae* [7]. To test the hypothesis that ComE1 from *P. multocida* could represent just one of a family of Fn/DNA-binding proteins, the homologous proteins from a further five members of the *Pasteurellaceae* were cloned and expressed as GST-fusion proteins (Table 2). These five recombinant fusion proteins were tested for their ability to bind to both Fn and pUC19 DNA in direct binding ELISAs. Recombinant ComE1 proteins from *H. influenzae*, *A. pleuropneumoniae*, *A. actinomycetemcomitans*, *M. haemolytica* and *M. succiniproducens* bound to both DNA (Fig. 7a) and Fn (Fig. 7b). The homologues from *M. haemolytica* and *M. succiniproducens* showed substantially less binding to both Fn and DNA compared with the proteins from *P. multocida*, *H. influenzae*, *A. pleuropneumoniae* and *A. actinomycetemcomitans* (Fig. 7). Competition ELISAs showed that, as is the case for ComE1 from *P. multocida*, all of these recombinant proteins bound to the 120 kD cell-binding domain of Fn (data not shown). K_D_ values for the interaction of these homologues with pUC19 were determined by surface plasmon resonance. These experiments were performed using the GST-free recombinant proteins (produced by cleavage of GST from the GST-fusion proteins) as there is evidence that the avidity effects that result from the dimerization of GST could result in overestimation of K_D_ values [16]. These experiments showed that the ComE1 proteins from *A. pleuropneumoniae* and *H. influenzae* had the lowest K_D_ values for the interactions with pUC19 DNA (Table 3). A much higher K_D_ value was determined for the interaction of the ComE1 protein from *M. succiniproducens* with pUC19 and no binding of the ComE1 protein from *M. haemolytica* to pUC19 was detected in these experiments (Table 3).

**Table 2:** Homologues of PM1665 (ComE1) from members of the Pasteurellaceae selected for cloning and expression.

Organism	ComE1 Gene	Homology to PM1665 (% Identity)	Size of predicted protein product
*H. influenzae*	*hi1008*	60	112
*A. pleuropneumoniae*	*APL_1406*	52	114
*A. actinomycetemcomitans*	*aa1426*	50	109
*M. haemolytica*	*mhORF35*	41	211
*M. succiniproducens*	*ms0826*	50	111

**Table 3:** K_D_ values for the interaction of recombinant ComE1 proteins with pUC19.

Recombinant ComE1 protein	K_D_ (µM)
PM1665	7.3
Hi1008	5
APL_1406	4
Aa1426	16
MhORF35	-
Ms0826	46

### Inactivation of *comE1* in A. pleuropneumoniae

**Figure 8:**
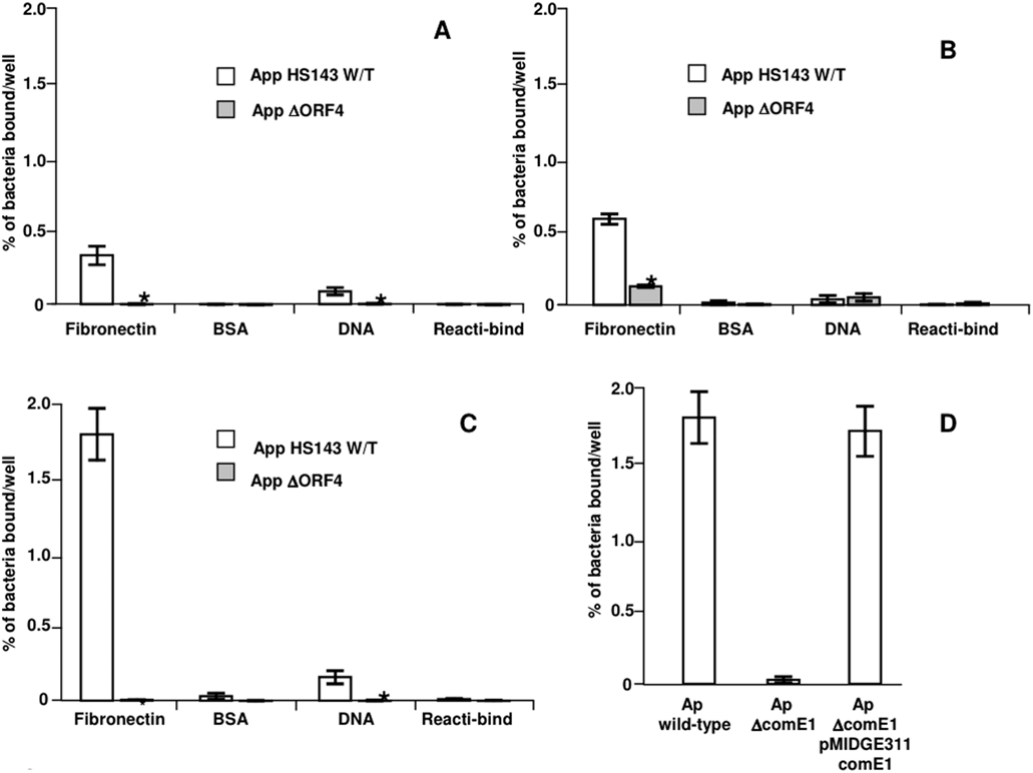
Binding of wild-type *A. pleuropneumoniae* or *ApΔcomE1* to Fn or pUC19 DNA. Bacteria were grown to early exponential phase (A), late exponential phase (B) or stationary phase (C) and tested for their ability to bind to wells coated with Fn or DNA. Asterisks indicate significant differences (independent t-test; see text for P values) in the binding of wild-type *A. pleuropneumoniae and ApΔcomE1* to each ligand. Restoration of binding of stationary phase *ApΔcomE1* to wells coated with Fn by provision of the *comE1* gene on plasmid pMIDGE311 (D).

**Figure 9:**
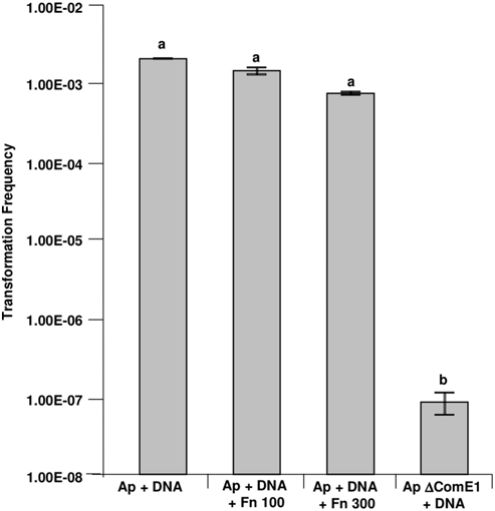
Comparison of the transformation frequency of wild-type *A. pleuropneumoniae* and *ApcomE1*. The effect of the presence of Fn on the transformation frequency of wild-type *A. pleuropneumoniae* was also tested. Bars with different letters are significantly different from each other (one-way ANOVA, P<0.001).

To determine the contribution of ComE1 to bacterial binding to Fn and DNA, the *A. pleuropneumoniae comE1* gene was insertionally inactivated. This approach was taken for two reasons: firstly, the availability of a naturally transformable strain of *A. pleuroneumoniae* [17], that is genetically more tractable than *P. multocida* and secondly, the fact that *A. pleuroneumoniae* is naturally competent allows us to investigate a possible role for ComE1 in natural transformation in this bacterium. The *comE1* gene was insertionally activated with a kanamycin gene (*Kan* ^R^) by alleleic replacement. The resultant mutant was verified by PCR (using primers 5′-ACAAGCGGTTTCACCCATTCGGGTTTCTACG-3′ and 5′-ACAAGCGGTGTAGTTTCAGTCGTAGGCGCTG-3′ anddesignated *ApΔcomE1*. Binding assays were used to compare the relative capacities of *A. pleuropneumoniae* HS143 and its isogenic *ΔcomE1* mutant, in various stages of growth, to bind to Fn and DNA. The growth phase made a significant difference to the ability of wild-type *A. pleuropneumoniae* to bind to Fn, with far greater numbers of bacteria binding when the bacteria were grown to the stationary phase (Fig. 8c) compared with either early (Fig. 8a) or late exponential phases (Fig. 8b). Wild-type *A. pleuropneumoniae* in the early exponential or stationary phases bound to DNA, but the numbers of bacteria binding to DNA in these growth phases were always much lower than those binding to Fn (Fig. 8). The loss of *comE1* resulted in a significant decrease (paired t-test; P<0.001) in the number of bacteria bound to Fn in all stages of growth. Complementation of *ApΔcomE1* with the *comE1* gene supplied on the plasmid pMIDG311 restored binding of *ApΔcomE1* to Fn at levels similar to that observed for wild-type *A. pleuropneumoniae* (Fig. 8d). Thus, we could rule out polar effects as an explanation of the change in binding of the *comE1* mutant. The effect of *comE1* inactivation on bacterial competence was also tested. There was a 10^4^-fold decrease in the ability of *ApΔcomE1* to undergo natural transformation compared with wild-type *A. pleuropneumoniae* (Fig. 9). As ComE1 recombinant protein bound to both Fn and DNA, the effect of the presence of soluble Fn on DNA uptake was also tested. There was no significant effect (one-way ANOVA) of Fn (at concentrations of up to 300 µg/ml) on transformation frequency in wild-type *A. pleuropneumoniae* (Fig. 9). Attempts were made compare the transformation frequencies of *ApΔcomE1* and the complemented mutant to that of wild-type *A. pleuropneumoniae* using donor DNA from a spontaneous streptomycin resistant mutant (Ap15*Str^R^*). However, for some reason the frequency of transformation of the wild-type strain HS143 to *Str^R^* was 4 log orders lower compared to the frequency of transformation to *Kan^R^* or *Chl^R^*, and no transformants were detected with either the *ApΔcomE1* or the complemented mutant using the *Str^R^* donor DNA.

## Discussion

We have recently discovered that *P. multocida* encodes a unique Fn-binding protein, PM1665, which binds with nanomolar affinity to a site in the cell binding Fn type III_9-10_ repeats of this glycoprotein, and acts as a major Fn adhesin for this organism. All other Fn-binding proteins and Fn adhesins bind either to the N- or C-terminal regions of this protein. Bioinformatics analysis reveals that PM1665/ComE1 has homologues in all members of the *Pasteurellaceae* where genome sequences are available, suggesting that this is an evolved family of Fn-binding proteins with similar biological roles. The unique site and high affinity of binding of the *P. multocida* ComE1 protein to Fn strongly suggested that binding to Fn was the major function of this class of protein. All of the ComE1 proteins studied here also have sequence homology with the C-terminal end of the ComEA protein from *Bacillus subtilis*, which is involved in DNA binding to the cell surface and uptake into cells [5], [18], [19]. We, therefore, asked whether PM1665, now designated ComE1 in line with the homologous protein in *H. influenzae* [8], bound to DNA? Initial experiments revealed that genomic DNA from *P. multocida* inhibited the binding of purified *P. multocida* ComE1 to Fn in a dose-dependent manner. This inhibition was replicated by pUC19, a plasmid which allowed us to calculate how much DNA we were adding as a competitor and showed that binding to DNA is independent of the presence of species-specific sequences such as USS. It was also demonstrated that *P. multocida* ComE1 bound directly to dsDNA but not to single stranded DNA. DNA binding was dependent on the size of the DNA fragments used in the assay, suggesting there were multiple sites for binding, as would be expected of a protein binding to DNA in a sequence-independent manner. Surface plasmon resonance, using a BIAcore 3000 instrument, was used to assess the affinity of binding and showed that the *P. multocida* ComE1 had a rapid on- and off-rate. It was possible to calculate the K_D_ value which was around 7 µM. This is around two log orders higher than the K_D_ of 100 nM for the binding of *P. multocida* ComE1 to Fn. It is assumed that the significant inhibition by DNA of the binding of *P. multocida* ComE1 to Fn is due to the fact that there are multiple binding sites in this double helix compared with only the two binding sites in the Fn glycoprotein. The Biacore experiments described here were used to give an indication of the stoichiometry of the interaction of ComE1 with pUC19 DNA. The maximal binding is related to the number of binding sites of the immobilised molecule, and the amount of ligand immobilised. A value of 120 was calculated for the stoichiometry for this interaction. This observation increases the effective concentration of binding sites in the DNA ELISA experiments by several orders of magnitude and so probably explains the differences we see in binding between fibronectin and DNA.

Using truncation mutants and synthetic peptides, we had previously found that the binding of *P. multocida* ComE1 to Fn required the participation of the two HHH motifs plus a small conserved run of amino acids (VNINTA) [1]. This region was the same segment of ComE1 required to bind to dsDNA, perhaps not surprisingly as HHH motifs are found in certain DNA-binding proteins [9], [10].

Is binding of the *P. multocida* ComE1 to DNA part of a DNA uptake mechanism linked to competence or does DNA binding play some other role such as in bacterial adhesion? We have shown that the binding of *P. multocida* to Fn is blocked by addition of *P. multocida* ComE1 and by a blocking monospecific antiserum raised to this protein in rabbits. In the present study we have shown that *P. multocida* also binds to 96 well plates coated with dsDNA. Moreover, this binding can be significantly inhibited by addition of recombinant *P. multocida* ComE1. Although we have shown that ComE1 from *P. multocida* is expressed on the cell surface and that the binding of this bacterium to Fn is blocked by recombinant *P. multocida* ComE1 and by an antiserum to this protein, the key experiment of inactivating the *comE1* gene had, despite multiple attempts, not been possible in our hands. However, we have been able to inactivate and complement the *comE1* gene in *A. pleuropneumoniae*. We tested the binding of *A. pleuropneumoniae* to Fn and DNA using cells in various stages of the bacterial growth cycle. This revealed that there were significant differences in bacteria at different stages of growth with regards to binding to Fn, and, to a lesser extent to DNA. Maximum binding was found with bacteria in stationary phase. There was only a very small amount of binding of bacteria to DNA and, again, this was greatest in stationary phase organisms. This inactivation of the *comE1* gene completely inhibited the binding of *A. pleuropneumoniae* to Fn and to DNA, and complementation of the gene returned binding to normal levels showing that this effect was due to the absence of the ComE1 protein and not to polar effects.

The homology of the *Pasteurellaceae* ComE1 proteins with the ComEA and ComE proteins known to be involved with bacterial competence has been noted. Bacterial competence is a physiological state which allows bacteria to bind and take up DNA from the environment, and natural transformation occurs when portions of the DNA are integrated into the chromosome via homologous recombination [19]. The conventional view is that DNA is taken up by bacteria for recombination [20], [21], but there is also evidence that the DNA may be used as a nutrient [22], [23], [24], [25]. What are the likely functions of these ComE1 proteins in the *Pasteurellaceae*? Natural transformation has only been demonstrated in three members of the *Pasteurellaceae*: *H. influenzae*, *A. pleuroneumoniae* and *A. actinomycetemcomitans* [13], [17], [26]. Competence in the *Pasteurellaceae* is under the control of a number of genes, all of which must presumably be fully functional in order to confer competence. Only the genomes of *H. influenzae*, *A. pleuropneumoniae*, *A. actinomycetemcomitans* and *P. multocida* have fully intact sets of competence genes, suggesting reasons why the other species may not be transformable [17]. In the present study we have now found that inactivation of the *comE1* gene in *A. pleuroneumoniae* serotype 15 (strain HS143) results in a 10^4^-fold decrease in the transformation frequency in this bacterium. Efficient uptake of DNA in *H. influenzae* and *A. pleuroneumoniae* is dependent upon the presence of a 9 base-pair sequence known as an ‘uptake signal sequence’. However, binding of ComE1 to DNA appears to be non-sequence specific, so the role of ComE1 in competence may be confined to binding of DNA molecules that are then selected for uptake by an, as yet unknown, sequence-specific receptor.

This is remarkably similar to the role of the ComE1 homologue (known as ComE) in *Neisseria gonorrhoeae*, where there is also direct experimental evidence for a role in competence for this protein. In contrast to the single copy of *comE1* in the *Pasteurellaceae*, there are four copies of *comE* in the *N. gonorrhoeae* genome and serial deletions resulted in decreases in transformation frequencies, with a reduction of 4×10^4^-fold reduction when all copies were deleted [6]. These authors also demonstrated that recombinant His-tagged ComE bound to DNA in a non-sequence specific manner [6] which is similar to the GST-fusion proteins tested here. As is the case for the competent members of the *Pasteurellaceae*, competence in the *Neisseriae* is also dependent upon a 10 bp DNA-uptake sequence, but the ComE protein which binds to DNA in a non-specific manner, is thus far the only competence factor that has been shown to interact with incoming DNA.

To summarise these results, we have identified a family of homologous proteins in the *Pasteurellaceae* which have homology to known non-specific DNA binding proteins in other organisms. The prototype, *P. multocida* ComE1 was initially identified as a high affinity adhesin for Fn binding by a unique mechanism. Other members of this protein family also bind Fn, although to different extents. Following up on the ComEA homology, we have discovered that all of these proteins also bind to DNA. The ComE1 protein from *P. multocida* is also involved in the binding of this bacterium to DNA. To further investigate the role of this protein we have inactivated the *comE1* gene in *A. pleuropneumoniae*. This resulted in: (i) the complete loss of the ability of the bacterium to bind to Fn and DNA and (ii) a four log order decrease in natural transformation compared to wild-type. Thus, in one protein we describe three distinct functions: (i) a fibronectin adhesin; (ii) a DNA adhesin and (iii) a role in natural transformation. This raises the question of whether these functions are linked. Fibronectin is a major eukaryotic protein found in all body fluids, on cell surfaces and in the extracellular matrices. It is a key host ligand for bacterial adhesion. Could fibronectin interfere with the uptake of DNA during transformation? The result of adding soluble Fn (to concentrations found in body fluids) to *A. pleuropneumoniae* cultures failed to inhibit natural transformation. However, it is possible that *in vivo* the Fn protein takes up some unique configuration on the surface of cells or in matrices that could interfere with DNA uptake and transformation. It would be very interesting to determine if any of the other known competence-related DNA-binding proteins, such as ComEA, also have Fn-binding activity.
